# Elastic straining of free-standing monolayer graphene

**DOI:** 10.1038/s41467-019-14130-0

**Published:** 2020-01-15

**Authors:** Ke Cao, Shizhe Feng, Ying Han, Libo Gao, Thuc Hue Ly, Zhiping Xu, Yang Lu

**Affiliations:** 1 Department of Mechanical Engineering, City University of Hong Kong, Kowloon, Hong Kong China; 2 Applied Mechanics Laboratory, Department of Engineering Mechanics and Center for Nano and Micro Mechanics, Tsinghua University, Beijing, 100084 China; 3 School of Mechano-Electronic Engineering, Xidian University, Xi’an, 710071 China; 4 Nano-Manufacturing Laboratory (NML), Shenzhen Research Institute of City University of Hong Kong, Shenzhen, 518057 China; 5 Department of Chemistry and Center of Super-Diamond & Advanced Films (COSDAF), City University of Hong Kong, Kowloon, Hong Kong China; 6 Department of Materials Science and Engineering, City University of Hong Kong, Kowloon, Hong Kong China

**Keywords:** Mechanical and structural properties and devices, Structural properties, Two-dimensional materials

## Abstract

The sp^2^ nature of graphene endows the hexagonal lattice with very high theoretical stiffness, strength and resilience, all well-documented. However, the ultimate stretchability of graphene has not yet been demonstrated due to the difficulties in experimental design. Here, directly performing in situ tensile tests in a scanning electron microscope after developing a protocol for sample transfer, shaping and straining, we report the elastic properties and stretchability of free-standing single-crystalline monolayer graphene grown by chemical vapor deposition. The measured Young’s modulus is close to 1 TPa, aligning well with the theoretical value, while the representative engineering tensile strength reaches ~50-60 GPa with sample-wide elastic strain up to ~6%. Our findings demonstrate that single-crystalline monolayer graphene can indeed display near ideal mechanical performance, even in a large area with edge defects, as well as resilience and mechanical robustness that allows for flexible electronics and mechatronics applications.

## Abstract (web summary)

The extraordinary mechanical properties of graphene are usually measured on very small or supported samples. Here, the authors develop a method to test a large area of graphene and show that even with edge defects it displays near-ideal mechanical performance.

## Introduction

Since graphene was studied by Geim and Novoselov through mechanical exfoliation from the highly oriented pyrolytic graphite in 2004^1^, it has been considered as an ideal material for facilitating thinner, faster electronic transistors, transparent touch screens, light panels, solar cells because of its near perfect two-dimensional (2D) crystal structure, high intrinsic strength, high transmittance, thermal conductivity, and electron mobility^2–7^. New methods were later developed to fabricate large-scale graphene monolayers, among them the most commonly used method is chemical vapor deposition (CVD), which can fabricate monolayer graphene towards mass production^6,8^. The mechanical robustness of them, however, is the basis for successful realization of their structural and functional performances. Theoretical investigations^4,9,10^ show that the Young’s modulus and ideal tensile strength of graphene are about 1 ± 0.1 TPa and 100–130 GPa, with maximum strains to failure up to ~13–19% and ~20–26% (with nonlinear elastic behavior at large strain^10–12^) along the armchair and zigzag directions, respectively. With the presence of defects (such as point defects, grain boundaries, edge defects) in large-area CVD-grown graphene, it is expected that the corresponding tensile strength shall be considerably reduced^7,10,13–17^. These understandings, however, remain to be assessed by direct experimental evidences.

Fracture properties of free-standing graphene with pre-crack, in forms of bilayer and multilayers, were experimentally measured in scanning electron microscope (SEM) and transmission electron microscope (TEM)^18–20^. These pre-cracked samples fracture in a brittle manner, featuring breaking stress significantly lower than the ideal strength of graphene. Still, direct measurement of the realistic strength of crack-free monolayer graphene or below the flaw-tolerance limit^21^ has not yet been made, which are more relevant for 2D material device applications based on high-quality single-crystalline graphene where cracks with lengths conforming to the applicability of linear elastic fracture mechanics do not show up (with only defects created during the growth and sample fabrication processes)^22,23^. Alternatively, indirect tests through nanoindentation into suspended graphene were earlier performed. Lee et al. reported that the intrinsic Young’s modulus of monolayer graphene can be indeed up to 1 TPa by the atomic forces microscope-based nanoindentation, and the intrinsic strength is up to 130 GPa, suggesting that graphene is the strongest material^5^. Consequently, the localized breaking stress measured by indenting graphene with grain boundaries is reduced by 15% from that of the pristine lattice^24^. However, these local tests measured only a small area of graphene under the indenter tip, and the strain distribution in the whole graphene membrane was highly non-uniform. This very sensitivity to the local atomic structures and prominent geometrical effects in indentation tests^25^ limit the technique in providing a reliable assessment of mechanical performance of large-area graphene with engineering relevance in practical applications, such as reinforced composites and electromechanical devices. Burst tests were recently carried out to directly measure the strength of suspended graphene under gas flow measurement, where a wide distribution of burst pressures was reported and attributed to wrinkles and defects^26^. As the engineering strength and strain to failure of a brittle material are controlled by the weakest point, a direct tensile test of large-area crack-free graphene membrane under uniaxial straining condition remains as the most efficient way for a quantitative study.

Besides, mechanical straining of graphene has been investigated as it can actually induce lattice distortion and phonons, modify the chemical reactivity and magnetic characteristics, and modulate their electronic and optoelectronic properties^27–31^. Recently, twisted bilayer graphene around the first magic angle (*θ* ≈ 1.1°) has been reported for the realization of unconventional superconductivity and correlated insulator behavior^32,33^. Alternatively, by varying the interlayer strain with hydrostatic pressure, superconductivity in twisted bilayer graphene can be also achieved^34^. However, significant strain-engineering effects often require large elastic strain to be applied, especially for monolayer graphene with zero intrinsic bandgap^31,35^. In contrast, in the experimental studies reported in literature, the stretchability of finite graphene sheets was limited by the presence of point and line defects, resulting in a common tensile strain to failure of ~1%^7,36^, far below the strain level associated with notable strain effects. These facts clearly indicate that a sample-wide elastic strain level much higher than 1% is desired to endow graphene with realistic strain-tunable device applications^27^.

In this work, we circumvent the gripping problem by developing well-controlled sample transfer and shaping techniques, namely well control of the shape of free-standing CVD-grown graphene samples, which were transferred onto the tensile straining stage through a modified wet-transfer method, and explore the in-plane mechanical responses of suspended monolayer graphene by our previously developed in situ nanomechanical testing platform under SEM^37^. We demonstrate their representative engineering tensile strength reaching ~50–60 GPa with sample-wide elastic strain up to ~6%, even in a large area with edge defects, displaying near-ideal mechanical performance, high resilience, and mechanical robustness.

## Results

### Transfer and characterization of graphene samples

**Fig. 1:**
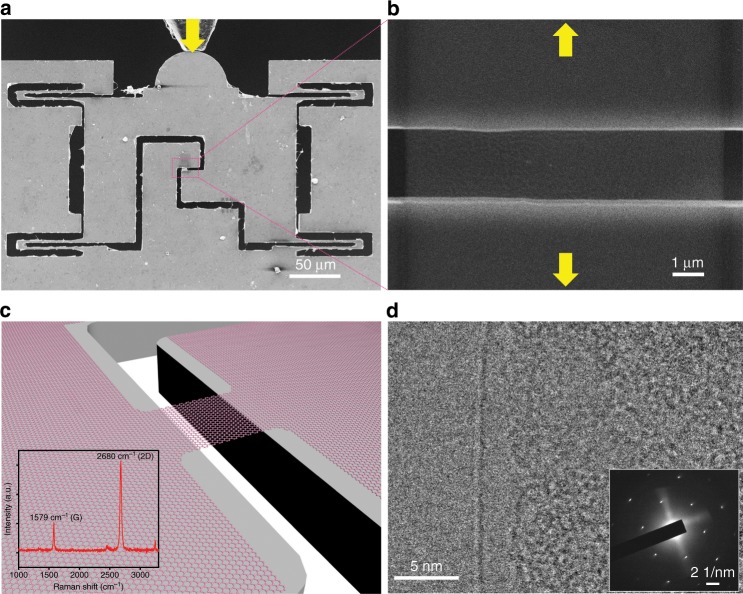
Experimental setup and characterization of free-standing graphene. **a** In situ scanning electron microscope (SEM) tensile testing of a single-crystalline suspended graphene sample based on a push-to-pull (PTP) micromechanical device actuated by an external quantitative pico-indenter, the yellow arrow indicates the indentation direction during a tensile testing process. **b** Zoom-in view of the pink rectangle area in **a** showing a suspended graphene ribbon sample, whereas the yellow arrows indicate the tensile loading direction. **c** Illustration of a free-standing graphene ribbon sample suspended between the device gap. The inset in **c** shows Raman characterization of the monolayer graphene sample, the ratio of 2D to G is ~3. **d** TEM characterization of the edge of the suspended sample, inset showing the single-crystalline SAED pattern.

To conduct in situ tensile tests, monolayer graphene samples were chosen and transferred onto a push-to-pull (PTP) micromechanical device through a polymethyl-methacrylate (PMMA)-assisted method as described in the Methods part. The graphene samples loaded on PTP devices were then cut into ribbon shape by focused ion beam (FIB) processing, with controlled size/area of the suspended part, as shown in Fig. 1a–b. Although outside the gap (ribbon) area, the whole micromechanical device was covered with the continuous monolayer graphene apart from the suspended sample area for firm fixing through van der Waals attraction^5^ to the substrate as illustrated in Fig. 1c. The corresponding yellow arrows denote the moving direction of the pico-indenter in Fig. 1a, to actuate the PTP device and record the force-displacement data, and the loading direction of the free-standing graphene sample in Fig. 1b, for tensile straining. The suspended graphene sample on the PTP device appears almost transparent owing to its monolayer nature. A typical Raman spectrum acquired from the transferred graphene on the gap is shown in the inset of Fig. 1c, the intensity ratio of the 2D (~2680 cm^−1^) and G (~1579 cm^−1^) peaks is ~3, and the D peak (~1345 cm^−1^) is negligible, indicating the transferred sample is monolayer^7^ of high quality. The edges of the suspended graphene were characterized separately inside a high-resolution transmission electron microscope operating at 80 kV. As can be seen from Fig. 1d, only one dark line in the edge again confirms the monolayer nature, and the corresponding selected area diffraction (SAED) pattern also shows only one set of hexagonal diffraction pattern. The crystalline structure of graphene ribbons suspended on the gap was further characterized by TEM multi-point diffraction analysis. From a series of SAED patterns as shown in Supplementary Fig. 1, the same diffraction pattern appeared from the edge to the center area, thus the single-crystalline nature of the tested samples can be confirmed.

### In situ tensile elastic straining of graphene

**Fig. 2:**
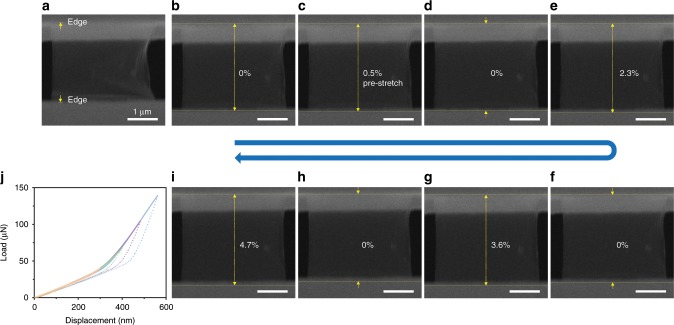
Fully recoverable elastic straining of a suspended graphene monolayer. **a**–**i** The sequence SEM images of the recorded video during the tensile testing procedure with the largest indentation displacement of 300 nm **a**–**c**, 400 nm **d**–**e**, 500 nm **f**–**g**, 600 nm **h**–**i**. The yellow arrows in **a** indicate the edges of the PTP device gap (tilted view). The first cycle **b**–**c** was to pre-stretch the relaxed free-standing single-crystalline graphene sample for marking the initial gauge length. The scale bar in all images are 1 μm. **j** The corresponding indenter load-displacement curves for cyclic elastic straining of the free-standing graphene (with unloading part marked in dash line).

To study the elastic properties and fracture behavior of monolayer graphene, displacement-controlled tensile tests were performed on the suspended graphene monolayers inside SEM, such as a sample shown in Fig. 2a (as well as Supplementary Fig. 2, showing the tested sample was pre-relaxed by releasing the prestress during FIB cutting). From the loading-unloading cyclic tensile straining process, the monolayer graphene demonstrates excellent elastic response, as shown in Supplementary Movie 1. During the first cycle, the pre-relaxed, draped graphene monolayer with possible ripples was pre-stretched to be fully tightened in Fig. 2b–c, marked as zero strain state. Then, it was loaded with increasingly larger indentation displacements with full recovery upon unloading in Fig. 2d–i, with the corresponding engineering elastic strains of 2.3%, 3.6%, and 4.7%, respectively. The corresponding loading/unloading-displacement curves in Fig. 2j for the elastic straining tests with increased displacement/strain amplitudes show good linearity and consistency among the four tensile cycles marked with different colors, indicating the high in-plane stretchability. It should be noted that, owing to the pre-relaxed state of the suspended sample, the initial overlapped load-displacement curves with a relatively lower slope refer that the indenter is actuating the PTP device only while the suspended graphene sample was not tightened and under actual loading (thus, the slope corresponds to the intrinsic stiffness of the PTP device). As the graphene ribbon became really pre-stretched in-plane until fully tighten, in the second stage, both graphene and the device bear the load and the linear loading-displacement curves with a higher slope capture the tensile mechanical behavior of free-standing graphene (although the slope/stiffness is contributed from both PTP device and graphene). During our tensile testing, the graphene samples undergo sample-wide uniform deformation, and the elastic strain can be well-controlled dynamically and continuously. We thus identified excellent tensile elasticity of CVD-synthesized single-crystalline graphene samples, up to ~5% fully recoverable sample-wide in-plane elasticity, which will enable considerable potential in the strain-engineering applications, such as lattice strain-controlled band structure engineering^28,31^.

### Ultimate stretchability and fracture strength

**Fig. 3:**
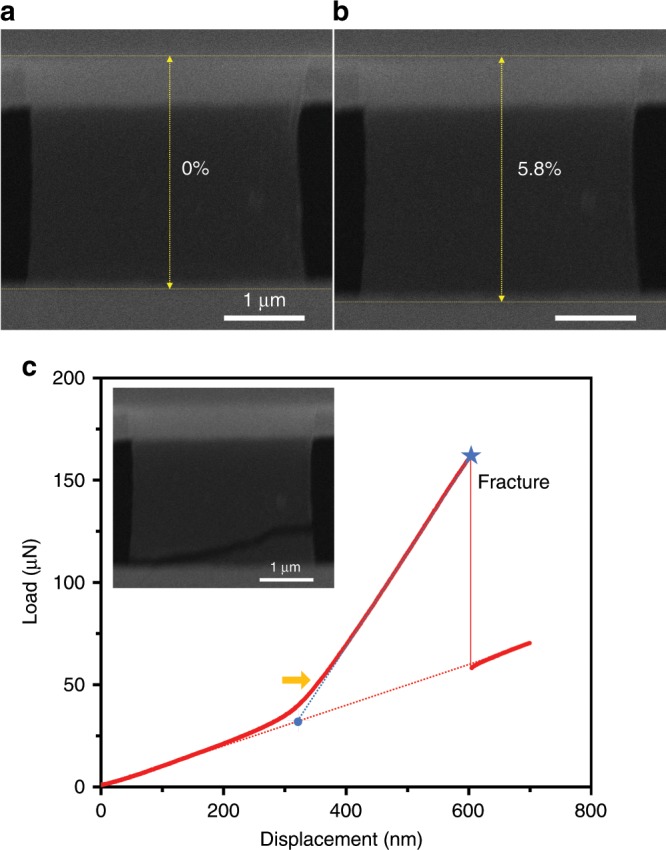
Tensile fracture of the free-standing graphene. **a** SEM image shows that the tested graphene sample under fully tightened state, with the scale bar shows 1 μm. **b** SEM image right before the tensile fracture, showing the peak strain state. **c** The corresponding indenter load-displacement curve, in which the blue dotted curve shows the linear fitting after the graphene sample being fully tightened and stretched (indicated with the orange arrow) while the inset shows the brittle fracture of the sample upon failure.

We further examined graphene sample by in situ tensile test until fracture. As shown in Fig. 3 and Supplementary Movie 2, the tested CVD graphene sample can be extensively stretched in-plane (Fig. 3a–b), with the peak engineering strain reaches ~5.8% without relaxation. The corresponding linear load-displacement curve in Fig. 3c upon graphene was fully tightened (as indicated by the orange arrow) indicate that the deformation was essentially pure elastic, followed by a typical brittle fracture (as shown in the insert of Fig. 3c). After failure (marked by the blue star), the indenter load instantly dropped and then increased again with the displacement control, whereas the slope follows the initial stage in which only the PTP device was actuated, as described above. Then, the actual load applied on the sample can be extracted by subtracting the contribution of the PTP device from the force-displacement data. The measured tensile stiffness before (blue dash line) and after the fracture of graphene (red dash line) in Fig. 3c is ~460 N/m and 110 N/m through linear fitting, respectively, thus the tensile stiffness of the free-standing graphene sample can be calculated around ~350 N/m. Accordingly, by assuming the whole sample is in a uniaxial stress condition, a 2D Young’s modulus of the graphene can be derived as *E*_2D_ =~309 N/m (see Methods), which is comparable with the 2D Young’s modulus of 348 N/m predicted from ab initio calculations^12^. The stress/strain state in the sample is not entirely uniform in our measurements, differing from the center free-standing part with zero transverse stress to the both clamping ends with transverse strain restricted (see Methods). However, to extract elastic constants from the force-displacement data measured in the experiments, assumptions have to be made. Our continuum mechanics simulations show that the uniaxial stress assumption is more reasonable than uniaxial strain that yields *E*_2D_ = 309 N/m, which applies for most area of the sample (see Supplementary Fig. 7). Using a nominal thickness of graphene of 0.335 nm^5^, a three-dimensional (3D) Young’s modulus of the free-standing graphene sample can be calculated as *E*_3D_ = ~920 GPa in the uniaxial stress assumption as well (see Methods), considering the actual width and gauge length of the tested sample. The measured Young’s modulus, along with some other sample results ranging from ~900–1000 GPa, is fairly close to the theoretical value of a pristine monolayer graphene crystal^4^, indicating the high quality of CVD-grown single-crystalline samples and that the presence of defects in the bulk region is negligible in graphene membrane tensile stiffness and modulus, even in large area.

## Discussion

**Fig. 4:**
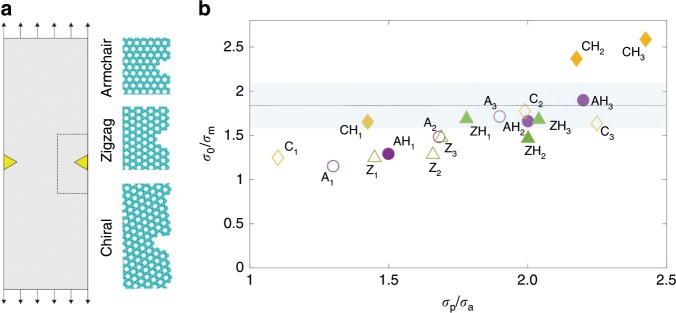
Molecular dynamics simulations on the fracture of free-standing graphene. **a** MD simulations of graphene ribbon under tensile tests. Atomic structures of the representative edge defects (dash box in the tensile specimen) are illustrated in the ball-stick models. **b** The stress ratios measured from the simulation data, where *σ*_0_ is the intrinsic tensile strength of ideal graphene lattice, *σ*_m_ is the simulation value measured for graphene ribbons with edge defects. *σ*_p_ and *σ*_a_ are the peak principal (tensile) stress and average stress in the graphene ribbons. The simulation data is labeled by the name of defects that are summarized in Supplementary Fig. 3. The blue shadowed area in **b** shows the corresponding ratio of our experimentally measured tensile strengths versus the ideal graphene strength *σ*_0_.

The corresponding engineering strength of the monolayer graphene in the case of Fig. 3, by taking peak tensile strain of ~5.8% before fracture, is calculated to be ~53 GPa. Compared with previous study on tensile fracturing of pre-cracked graphene^18^, our result clearly demonstrates much higher elastic stretchability and tensile strength for free-standing graphene without crack, suggesting the good usability of high-quality CVD-grown single-crystalline graphene. Nevertheless, the representative tensile fracture strength of ~50–60 GPa measured from our multiple samples is considerably reduced compared with the ideal strength of monolayer graphene (~100–130 GPa)^7^, which should be partly attributed to the edge defects implanted during the sample cutting process by FIB. To quantify this effect, we carried out atomistic simulations to measure the fracture strength of graphene ribbons with armchair, zigzag, and chiral edges under end-clamped tension (Fig. 4a). We created different types of edge defects according to the recent studies of ion irradiation damage in graphene (Supplementary Fig. 3)^38,39^. The corresponding fracture strengths are summarized in Fig. 4b. From the simulation results, we find that the ratio *σ*_0_/*σ*_m_ between the ideal strength of graphene (*σ*_0_) and the measured engineering tensile strengths of graphene ribbons with edge defects (*σ*_m_) is close to our experimental measurements, as shown in the shadowed area in Fig. 4(b), indicating that the FIB-induced defects at graphene edges are indeed detrimental for the mechanical resistance. As the concentration of edge defects can be reduced by optimizing the FIB or other sample cutting process, the engineering strength of free-standing single-crystalline graphene could be further increased. On the other hand, although significantly reduced, our experimentally measured sample-wide strength is still quite comparable to the intrinsic strength value from local measurement^5^, exceeding one-half of the ideal strength of graphene (i.e., deep ultra-strength^37^), which implies that the defects created by FIB cutting are localized at the edge region, and the contribution of edge defects (rather than crack) toward the graphene membrane strength and toughness is less significant. It should be noted that the presence of large amount of internal and edge defects across the whole sample area would obviously modulate the crack behavior and fracture toughness, such as leading to multiple crack stages^17^, whereas in our high-quality single-crystalline monolayer samples with mostly edge defects, the effect appears less considerable. At last, we calculated the average atomistic principal stress in the region away from defect is marked as *σ*_a_, and the peak principal stress identified near the defect is marked as *σ*_p_. The simulation results show that although the value of *σ*_p_ characterize the local stress state of graphene, the ratio *σ*_p_/*σ*_a_ displays a much wider span, indicating that the maximum principal tensile stress may not be a reliable indicator to determine the nucleation of fracture in single-crystalline graphene. This could be attributed to the significant anisotropy in fracture toughness of the hexagonal lattice that should be included in defining the criterion for failure^40^. Besides, in view of the likelihood of the presence of small in-plane misalignment of the tensile loaded sample between the two clamping ends, atomistic simulations are carried out to study the fracture strain and strength predicted for a free-standing graphene sample loaded with a non-uniform displacement field (Supplementary Fig. 6). The results showing that the tensile strength and strain to failure measured experimentally are close to simulation results imply that the effect of in-plane misalignment is minor.

Tensile fracture mechanism (i.e., crack initiation and propagation) of monolayer single-crystalline graphene was explored by the atomistic simulations coupled with TEM analysis on the fractured samples. The simulation results summarized in Supplementary Fig. 4 show that atomistic principal stress maximizes in the region near the clamping ends since the lattice straining is constrained at the interface between free-standing and clamped graphene parts. As a result, crack nucleates from the clamping ends if there are no edge defects, considering the low aspect ratio of the loading configuration. As the edge defects are inevitably introduced, the combined effects of stress concentration at the edges and clamping ends will lead to fracture initialization from the edges near the clamping ends, aligning well with our experimental evidences (Fig. 3 and Supplementary Movie 2). The crack propagation direction is analyzed through the post-mortem TEM investigation on the cleaved edges of a fractured monolayer sample with a pre-crack near the edge (for easier tracking). The tensile fracture process of this sample is shown in Supplementary Fig. 5 and Supplementary Movie 3. Both zigzag and armchair directions are identified (Supplementary Fig. 5d–e) upon post TEM analysis. Recent atomistic simulation studies suggest that in the scenario of Griffith fracture criterion with anisotropic edge energy densities included, the patterns of cleaved edges can be determined by the relative orientation between the loading direction and graphene lattice^41^. For a single-crystal graphene, except for a very narrow interval of strain direction at which mixed-direction cracks are favored, the armchair and zigzag cleaved edges are highly preferred for the fracture, which well explains our experimental findings on the crack path of free-standing monolayer graphene.

In summary, by developing robust sample transfer, shaping and clamping techniques, in situ quantitative tensile test of free-standing single-crystalline graphene monolayers has been successfully achieved, showing near-ideal tensile stiffness and elastic modulus (~900–1000 GPa) with representative engineering tensile strength as large as ~50–60 GPa, despite that the reduction in strength arises mainly from edge defects. These results demonstrate the potential of large-area CVD-grown single-crystalline graphene^8,42^ in practical engineering applications such as flexible electronics and ultra-strength composites^7^. Brittle fracture of the free-standing graphene initiated from the damaged lattice sites at edges near the clamping ends, whereas the ultimate stretchability and strength could be further optimized by controlling the edge states. This study reaffirms the high mechanical resilience of graphene, beyond the limit of local probe, and the measured sample-wide strain up to ~6% without inelastic relaxation brings the widely proposed strain engineering into practice^27^. At last, the in situ nanomechanical straining strategy we developed here for monolayer graphene, allowing for uniform, reversible and well-controllable strain modulation, provides opportunities for dynamically strain-tuned electronics and optoelectronics device applications of other 2D materials and their superlattices/heterostructures through elastic strain engineering^28,31,34,43^.

## Methods

### Transfer and sample preparation for tensile tests

CVD-synthesized monolayer graphene films on copper foil (from Sigma Aldrich) with PMMA coating (thickness < 100 nm) were transferred to the PTP device through an optimized chemical etching method. First, to release the graphene from the copper foil, the graphene with copper foil was immersed into Cu etchant (from Sigma Aldrich). Second, the graphene with PMMA was fished up with a piece of polyethylene terephthalate and released into distilled water for three times to remove Cu etchant thoroughly. Third, the graphene with PMMA coating was fished up with the PTP device and then left overnight in a dry cabinet so that the graphene attached firmly with the PTP device. Fourth, the PMMA was dissolved by acetone solution in a critical point dryer, which was used to reduce the surface tension of acetone during dissolving the PMMA layer and protect the suspended graphene on the gap of PTP device. Finally, the suspended graphene was cut into ribbon shape (e.g., width ~3.4 μm, gauge length ~3.0 μm for the sample in Figs. 2 and 3) by FIB using small current to reduce the ion beam-induced damage for samples, whereas the remaining part covering the whole PTP device surface to provide robust clamping for tensile testing. Graphene samples were characterized with Raman spectroscopy^44^ by a 514-nm laser, the laser power was set as 1.7 mW to prevent thermal heating and damage of graphene. TEM characterization of the graphene samples before and after testing was carried out using a JEOL 2100 F TEM.

### In situ SEM tensile testing

Graphene on the PTP device was tested using a Hysitron pico-indenter (PI85) inside a FEI Quanta 450 SEM^37^. The PTP device loaded with graphene was firstly mounted onto the PI85 testing platform, then the PI85 was mounted into the SEM chamber for in situ tensile testing and controllable straining. The load and displacement were recorded with the indenter transducer, whereas videos were recorded in situ by SEM (operating at 5–20 kV to reduce the electron beam effect). Tensile strain (*ε*) can be well controlled and measured directly from the SEM image sequences/videos. The tensile stiffness of the sample can be calculated by subtracting the inherent stiffness of the PTP device from indenter load-displacement curves, and the 2D and 3D Young’s modulus can be calculated as $E_{2{\mathrm{D}}} = k\frac{l}{W}$, $E_{3{\mathrm{D}}} = k\frac{l}{{tW}}$, where *k*, *t, l*, and *W* are the tensile stiffness, the thickness (taken as 0.335 nm for monolayer graphene), the gauge length and the width of the sample, respectively^20^. It should be note that to interpret the measured stiffness of the whole sample in terms of the Young’s modulus *E* as a material parameter, assumptions have to be made for the stress/strain state in the sample. In our experimental setup, the end-clamped loading condition enforces a uniaxial strain state at the ends. However, the center suspended part is free of transverse load. As a result, for samples with a sufficiently large aspect ratio, the uniaxial stress assumption ($\sigma _2 = 0$, 2 is the transverse direction) is more reasonable than the uniaxial strain assumption $\left( {\varepsilon _2 = 0} \right)$ for most area of the sample. We validated this argument by performing finite element methods-based simulations (Supplementary Fig. 7), and consequently grant $E_{2{\mathrm{D}}} = k\frac{l}{W}$ instead of $C_{11} = k\frac{l}{W}$, where *C*_11_ is the first element of the stiffness matrix C. For completeness, we also extracted the Young’s modulus in the uniaxial strain assumption, which yields $E_{2{\mathrm{D}}} = \frac{{\left( {C_{11}^2 - C_{12}^2} \right)}}{{C_{11}}} = 340\;{\mathrm{N}}/{\mathrm{m}}$, using $\nu = \frac{{C_{12}}}{{C_{11}}} = 0.169$ as reported^12^. Tensile stress (*σ*) applied on the free-standing graphene sample is then calculated through conventional $\sigma = E_{3{\mathrm{D}}} \times \varepsilon$ where a nominal thickness of 0.335 nm is used to convert *E*_2D_ into *E*_3D_.

### Molecular dynamics (MD) simulation

We constructed atomistic models of free-standing graphene ribbons with a periodic boundary condition (PBC) along the loading direction, and two free boundary conditions in the width and out-of-plane dimensions. The length and width of ribbon are 20 nm and 10 nm, which were validated to exclude size effects on the conclusions we made. Defects were created symmetrically at the ribbon edges, as illustrated in Fig. 4. Both bare and hydrogen-terminated edges were considered in the models (Supplementary Fig. 3). MD simulations were performed using the large-scale atomic/molecular massively parallel simulator package^45,46^. The adaptive intermolecular reactive empirical bond-order (AIREBO) potential function is used to describe the inter-atomic interaction in graphene^47^. For the parameters in AIREBO potential functions, the cutoff distance was adjusted to be 0.2 nm to avoid the spurious strengthening effect after structural failure^48^. The simulation started at 0 K and the temperature rise during tensile tests is on the order of 10 K. The effect of thermal fluctuation on mechanical responses is thus negligible. Uniform tensile strain was applied by deforming the simulation box and applying atomic displacements at a loading rate of 1 ns^−1^ before the atoms were allowed to move following their equations of motion. The atomic displacement was determined by assuming an affine deformation of the lattice, which is reasonable owing to the single-crystalline nature of our experimental samples. MD simulations at lower loading rates were also performed and compared with to verify that the deformation and fracture behavior are not affected by these dynamical effects. Tensile tests to study crack nucleation and propagation in clamped samples were carried out with two rows of aromatic rings at the clamping ends fixed or displaced (at a constant rate of 1 ns^−1^) in the simulations (Supplementary Fig. 4). The atomic virial stress was calculated, mapped to a two-dimensional regular mesh for analysis of the local stress field in the pristine and defective regions, and averaged over the sample to measure the tensile stress.

## Supplementary information

Supplementary Information

Description of Additional Supplementary Files

Supplementary Movie 1

Supplementary Movie 2

Supplementary Movie 3

## Data Availability

The data that support the findings of this study are available from the corresponding author upon reasonable request.

## References

[1] Novoselov KS (2004). Electric field effect in atomically thin carbon films. Science.

[2] Kelly, B. T. *Physics of Graphite*. (Applied Science, London, 1981).

[3] Geim AK, Novoselov KS (2007). The rise of graphene. Nat. Mater..

[4] Liu F, Ming PM, Li J (2007). Ab initio calculation of ideal strength and phonon instability of graphene under tension. Phys. Rev. B.

[5] Lee C, Wei X, Kysar JW, Hone J (2008). Measurement of the elastic properties and intrinsic strength of monolayer graphene. Science.

[6] Novoselov KS (2012). A roadmap for graphene. Nature.

[7] Papageorgiou DG, Kinloch IA, Young RJ (2017). Mechanical properties of graphene and graphene-based nanocomposites. Prog. Mater. Sci..

[8] Li X (2009). Large-area synthesis of high-quality and uniform graphene films on copper foils. Science.

[9] Zhao H, Min K, Aluru NR (2009). Size and chirality dependent elastic properties of graphene nanoribbons under uniaxial tension. Nano Lett..

[10] Wei Yujie, Yang Ronggui (2018). Nanomechanics of graphene. National Science Review.

[11] Cadelano E, Palla PL, Giordano S, Colombo L (2009). Nonlinear elasticity of monolayer graphene. Phys. Rev. Lett..

[12] Wei, X. D., Fragneaud, B., Marianetti, C. A., Kysar, J. W. Nonlinear elastic behavior of graphene: ab initio calculations to continuum description. *Phys. Rev. B* **80**, 205407 (2009).

[13] Zhang J, Zhao J, Lu J (2012). Intrinsic strength and failure behaviors of graphene grain boundaries. ACS Nano.

[14] Zhang T, Li XY, Gao HJ (2015). Fracture of graphene: a review. Int J. Fract..

[15] Guin Laurent, Raphanel Jean L., Kysar Jeffrey W. (2016). Atomistically derived cohesive zone model of intergranular fracture in polycrystalline graphene. Journal of Applied Physics.

[16] Shekhawat A, Ritchie RO (2016). Toughness and strength of nanocrystalline graphene. Nat. Commun..

[17] Dewapriya MAN, Meguid SA (2018). Tailoring fracture strength of graphene. Comp. Mater. Sci..

[18] Zhang P (2014). Fracture toughness of graphene. Nat. Commun..

[19] Wei XL (2015). Comparative fracture toughness of multilayer graphenes and boronitrenes. Nano Lett..

[20] Jang B (2017). Uniaxial fracture test of freestanding pristine graphene using in situ tensile tester under scanning electron microscope. Extrem. Mech. Lett..

[21] Gao HJ (2003). Materials become insensitive to flaws at nanoscale: lessons from nature. Proc. Natl Acad. Sci. USA.

[22] Bunch JS (2007). Electromechanical resonators from graphene sheets. Science.

[23] van der Zande AM (2010). Large-scale arrays of single-layer graphene resonators. Nano Lett..

[24] Lee GH (2013). High-strength chemical-vapor-deposited graphene and grain boundaries. Science.

[25] Song Z (2015). Defect-detriment to graphene strength is concealed by local probe: the topological and geometrical effects. ACS Nano.

[26] Wang L (2017). Single-layer graphene membranes withstand ultrahigh applied pressure. Nano Lett..

[27] Li J, Shan ZW, Ma E (2014). Elastic strain engineering for unprecedented materials properties. MRS Bull..

[28] Yu DP, Feng J, Hone J (2014). Elastically strained nanowires and atomic sheets. MRS Bull..

[29] Levy N (2010). Strain-induced pseudo-magnetic fields greater than 300 tesla in graphene nanobubbles. Science.

[30] Bissett MA (2013). Enhanced chemical reactivity of graphene induced by mechanical strain. ACS Nano.

[31] Si C, Sun Z, Liu F (2016). Strain engineering of graphene: a review. Nanoscale.

[32] Cao Y (2018). Unconventional superconductivity in magic-angle graphene superlattices. Nature.

[33] Cao Y (2018). Correlated insulator behaviour at half-filling in magic-angle graphene superlattices. Nature.

[34] Yankowitz M (2019). Tuning superconductivity in twisted bilayer graphene. Science.

[35] Pereira VM, Castro Neto AH, Peres NMR (2009). Tight-binding approach to uniaxial strain in graphene. Phys. Rev. B.

[36] Polyzos I (2015). Suspended monolayer graphene under true uniaxial deformation. Nanoscale.

[37] Zhang H (2016). Approaching the ideal elastic strain limit in silicon nanowires. Sci. Adv..

[38] Lehtinen O, Kotakoski J, Krasheninnikov AV, Keinonen J (2011). Cutting and controlled modification of graphene with ion beams. Nanotechnol.

[39] Buchheim J, Wyss RM, Shorubalko I, Park HG (2016). Understanding the interaction between energetic ions and freestanding graphene towards practical 2D perforation. Nanoscale.

[40] Hossain MZ (2018). Anisotropic toughness and strength in graphene and its atomistic origin. J. Mech. Phys. Solids.

[41] Kim K (2012). Ripping graphene: preferred directions. Nano Lett..

[42] Jin S (2018). Colossal grain growth yields single-crystal metal foils by contact-free annealing. Science.

[43] Wang SS (2016). Atomically sharp crack tips in monolayer MoS_2_ and their enhanced toughness by vacancy defects. ACS Nano.

[44] Ferrari, A. C. et al. Raman spectrum of graphene and graphene layers. *Phys. Rev. Lett.* 97, (2006). 10.1103/PhysRevLett.97.187401 17155573

[45] Plimpton S (1995). Fast parallel algorithms for short-range molecular-dynamics. J. Comput. Phys..

[46] Zhang WS (2017). SCStore: Managing scientific computing packages for hybrid system with containers. Tsinghua Sci. Technol..

[47] Brenner DW (2002). A second-generation reactive empirical bond order (REBO) potential energy expression for hydrocarbons. J. Phys. Condens. Mat..

[48] Xu ZP (2009). Graphene nano-ribbons under tension. J. Comput. Theor. Nanosci..

