# Supplementary material for: Elastic straining of free-standing monolayer graphene
Source: Nat Commun. 2020 Jan 15;11:284. doi: 10.1038/s41467-019-14130-0 (PMC6962388; doi:10.1038/s41467-019-14130-0)
Supplement: Supplementary file 1 — Supplementary Information [file 41467_2019_14130_MOESM1_ESM.pdf]

Supplementary Information

Elastic Straining of Free-Standing Monolayer Graphene

Cao et al.

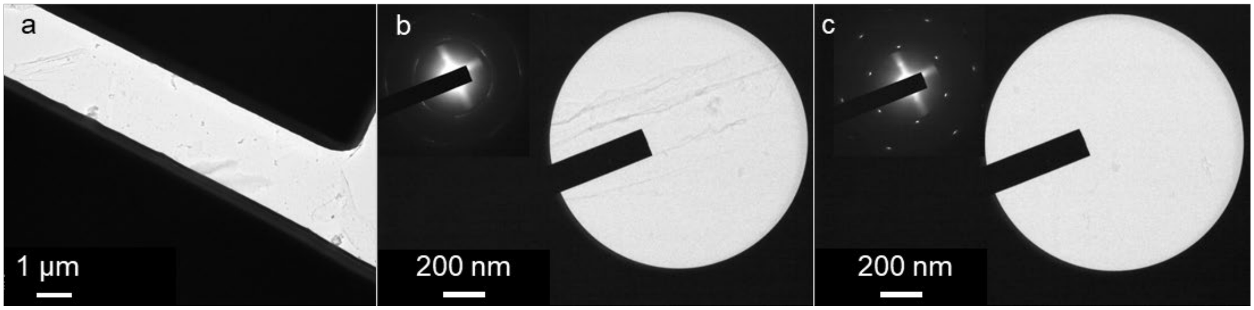

Supplementary Figure 1 Crystalline structures of a free-standing monolayer graphene on a PTP device: (a) low-magnification TEM image of the suspended graphene; (b, c) corresponding SAED patterns for the edges and the center area of the graphene, respectively.

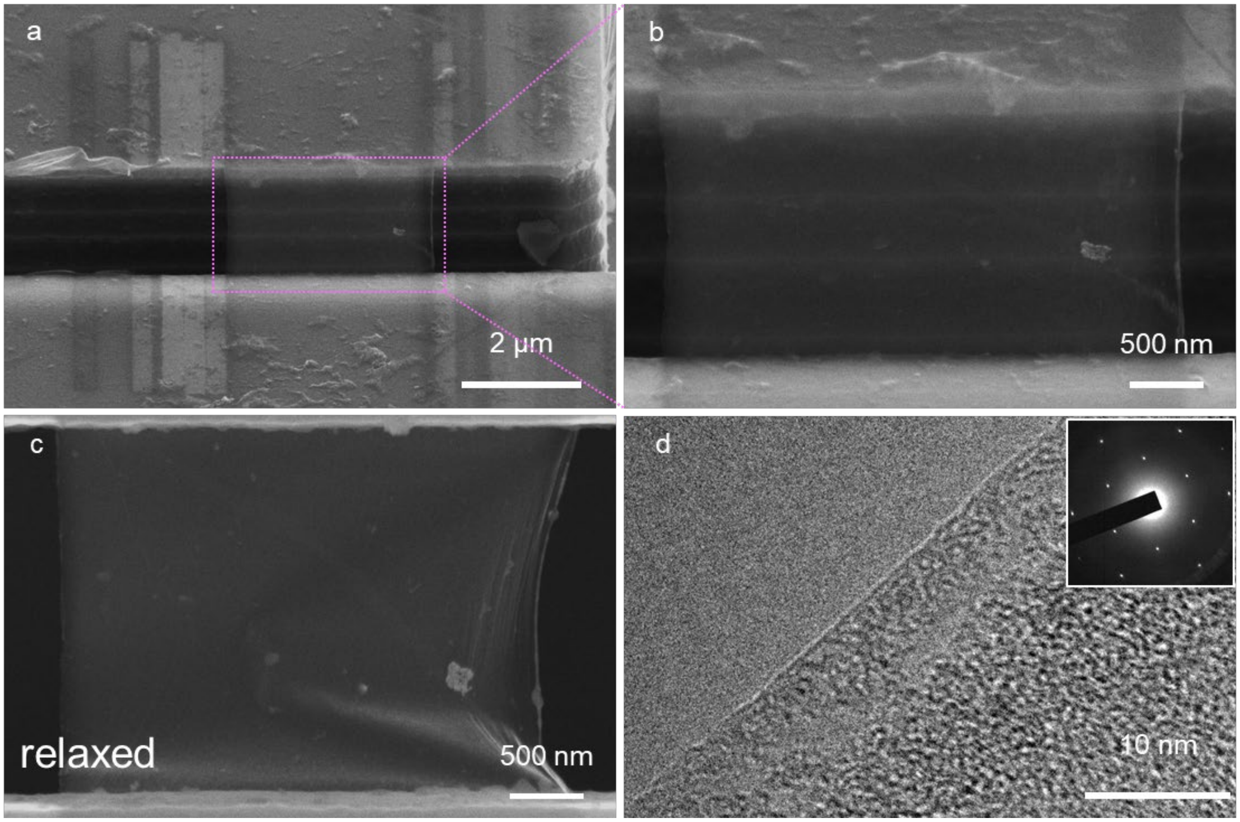

Supplementary Figure 2 The tested monolayer graphene sample on PTP device after focused ion beam (FIB) cutting: (a, b) low- and high-magnification SEM images of the suspended graphene under pre-stressed state; (c) the suspended graphene in the red rectangle area of (a) under relaxed state after the prestress was released; (d) TEM image of the sample edge and the insert SAED image show the monolayer single-crystalline character of the sample.

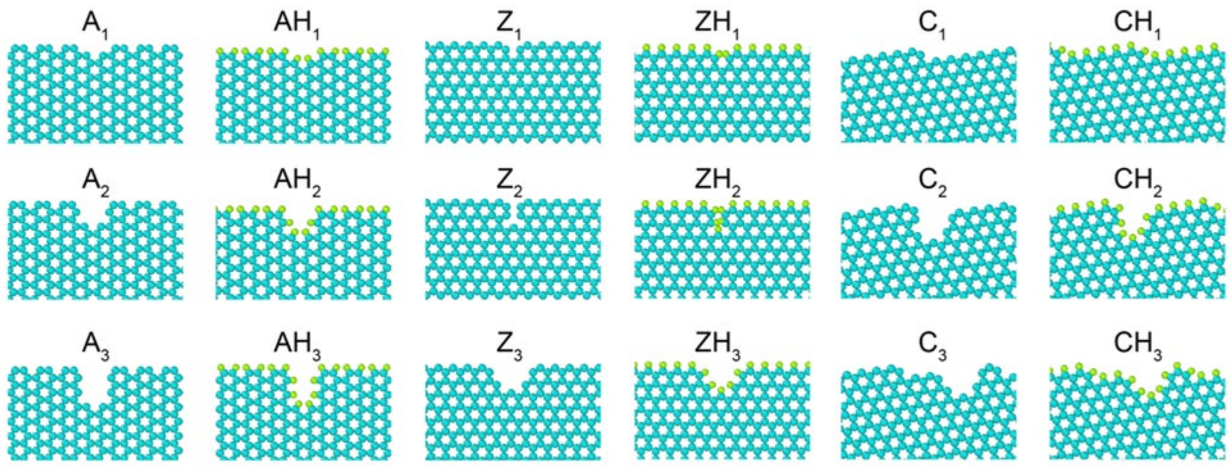

Supplementary Figure 3 Atomic structures of graphene edges with possible FIB-induced defects, where ‘A’, ‘Z’ and ‘C’ denote ‘armchair’, ‘zigzag’ and ‘chiral’, respectively. ‘H’ indicates edge hydrogenation. In our models, a (5,1) chiral edge is used without loss of generality.

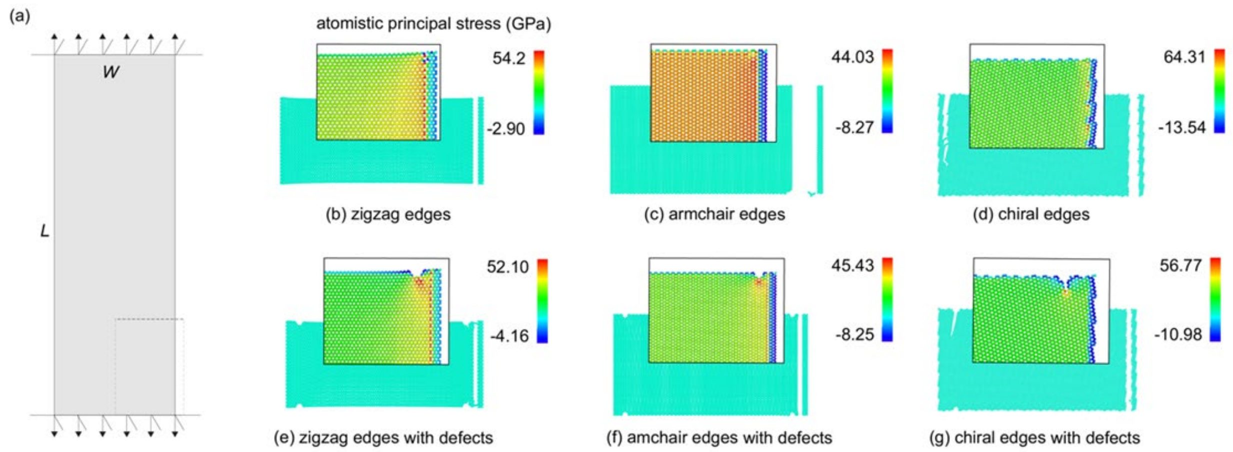

Supplementary Figure 4 Tensile testing simulations showing the nucleation of crack near the graphene clamping ends. For edge defect-free samples, the crack often nucleates at the clamping boundary since lattice deformation under clamping is more constrained there, resulting in reduced strength. With the presence of edge defects, the principal stress concentration at the defective sites would induce tensile fracture therein.

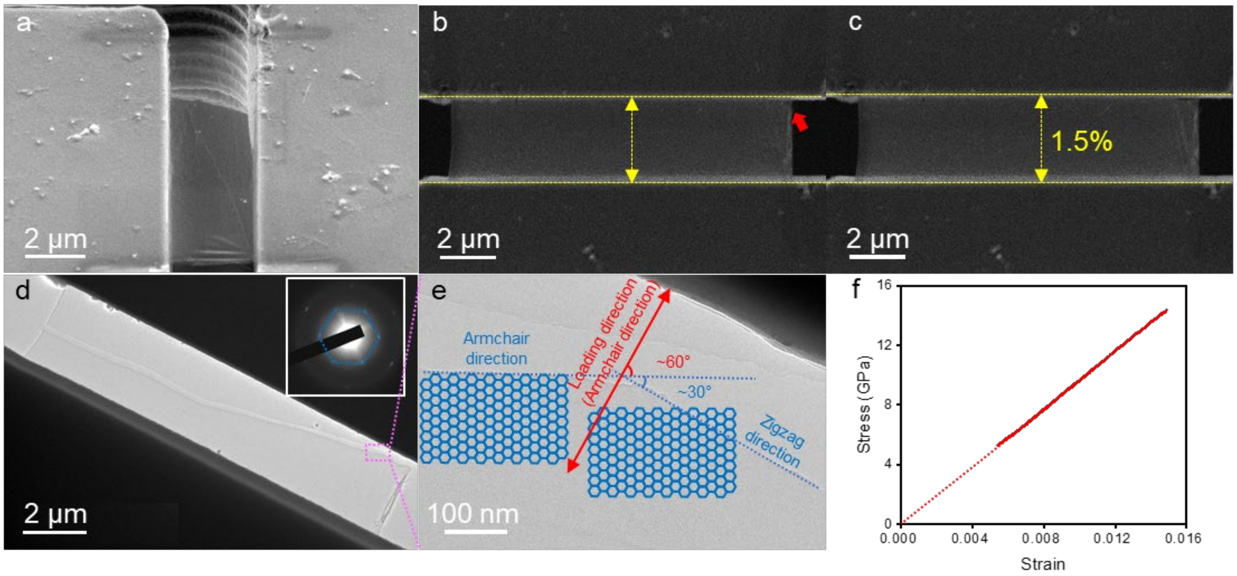

Supplementary Figure 5 Tensile fracture and crack propagation characterization of another single-crystalline graphene monolayer with a pre-crack (for better tracking the crack growth during in situ tension): (a) the pristine sample after FIB cutting; (b) SEM image of the sample when it is fully stretched. There is a visible crack before the test, as indicated by the red arrow; (c) SEM image of the sample right before fracture/cleavage. The measured strain is ~1.5%; (d, e) fracture morphology and crack directions of the fractured sample analyzed by TEM; (f) corresponding stress-strain curve of the pre-cracked monolayer graphene during elastic straining until fracture.

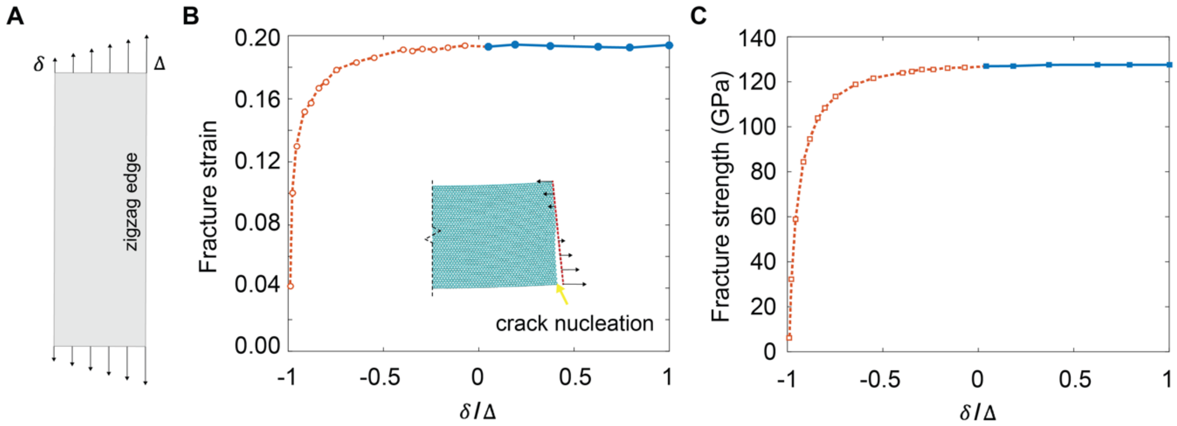

Supplementary Figure 6 Fracture strain and strength predicted for a free-standing graphene sample loaded in a misaligned configuration. (a) The clamping ends of a defect-free graphene sample are loaded with a linear displacement field (from δ to Δ). The predicted values of the (b) fracture strain and (c) fracture strength summarized as a function of the ratio δ/Δ. The results show that in our tensile tests with $\delta/\Delta > 0$, the effect of in-plane misalignment is minor.

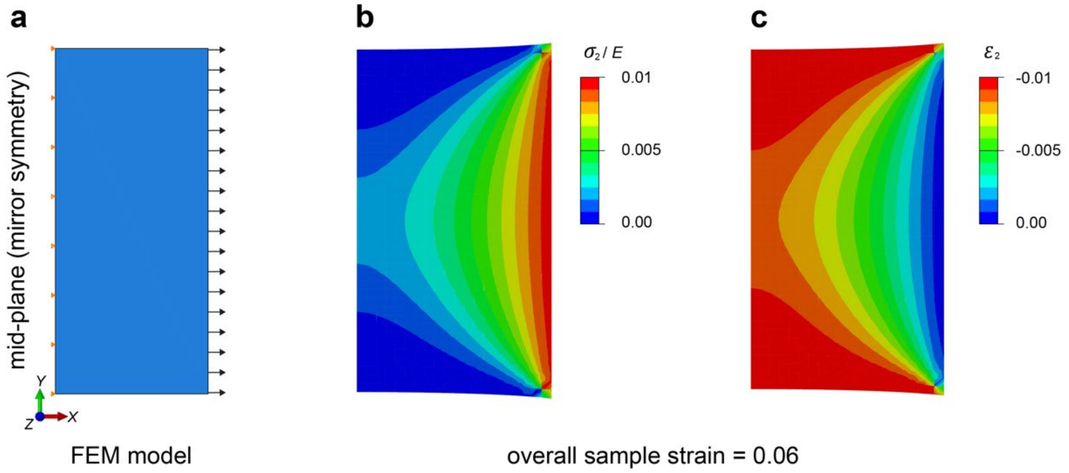

Supplementary Figure 7 Normalized transverse stress and strain distribution predicted through finite element methods (FEM)-based simulations. (a) Simulation set-up for a model of monolayer graphene following the sample geometry shown in Fig. 3. A mirror-symmetry boundary condition was used for the mid-plane (only half of the sample is illustrated), and a displacement-based loading condition was applied to the clamped end to stretch the sample. The material parameters E_{2D} = 348 N/m, v = 0.169 were chosen based on the theoretical predictions in Ref. 12. (b, c) Distributions of normalized transverse stress σ_2/E and transverse strain ε_2 in the sample, the comparison of which suggests that the uniaxial stress ($\sigma_2 = 0$) is a better assumption that uniaxial strain ($\varepsilon_2 = 0$) for most area of the sample.
