# Supplementary material for: Elastic straining of free-standing monolayer graphene
Source: Nat Commun. 2020 Jan 15;11:284. doi: 10.1038/s41467-019-14130-0 (PMC6962388; doi:10.1038/s41467-019-14130-0)
Supplement: Supplementary file 2 — Description of Additional Supplementary Files [file 41467_2019_14130_MOESM2_ESM.docx]

**Description of Additional Supplementary Files**

**Title: Supplementary Movie 1:**

Cyclic tensile straining of a free-standing monolayer graphene sample (64× speed).

**Title: Supplementary Movie 2:**

in situ Tensile testing of the graphene sample until fracture (64× speed).

**Title: Supplementary Movie 3:**

Tensile fracture of another monolayer graphene sample with a pre-crack (16× speed).
